# Dendritic Slow Dynamics Enables Localized Cortical Activity to Switch between Mobile and Immobile Modes with Noisy Background Input

**DOI:** 10.1371/journal.pone.0024007

**Published:** 2011-09-08

**Authors:** Hiroki Kurashige, Hideyuki Câteau

**Affiliations:** RIKEN BSI-TOYOTA Collaboration Center, RIKEN, Wako-shi, Saitama, Japan; University of Pittsburgh, United States of America

## Abstract

Mounting lines of evidence suggest the significant computational ability of a single neuron empowered by active dendritic dynamics. This motivates us to study what functionality can be acquired by a network of such neurons. The present paper studies how such rich single-neuron dendritic dynamics affects the network dynamics, a question which has scarcely been specifically studied to date. We simulate neurons with active dendrites networked locally like cortical pyramidal neurons, and find that naturally arising localized activity – called a bump – can be in two distinct modes, mobile or immobile. The mode can be switched back and forth by transient input to the cortical network. Interestingly, this functionality arises only if each neuron is equipped with the observed slow dendritic dynamics and with in vivo-like noisy background input. If the bump activity is considered to indicate a point of attention in the sensory areas or to indicate a representation of memory in the storage areas of the cortex, this would imply that the flexible mode switching would be of great potential use for the brain as an information processing device. We derive these conclusions using a natural extension of the conventional field model, which is defined by combining two distinct fields, one representing the somatic population and the other representing the dendritic population. With this tool, we analyze the spatial distribution of the degree of after-spike adaptation and explain how we can understand the presence of the two distinct modes and switching between the modes. We also discuss the possible functional impact of this mode-switching ability.

## Introduction

A dendrite of a pyramidal neuron has been observed in vitro to evoke localized large depolarization in response to a strong synaptic drive [1], [2]. This localized depolarization is called a dendritic spike. The observation of a dendritic spike has been becoming common [3]–[15], and most recently in vivo evidence has been found suggesting the presence of the dendritic spike in response to a sensory stimulus [16]. The widespread observations motivate serious computational study of its functional significance in the information processing in the brain. Poirazi et al. (2003) pointed out that a single pyramidal neuron with active dendrites behaves as a two-layer neural network [17], [18]. A collection of dendritic branches is regarded to work as *the first layer* since each branch performs local summation and produces near-digitized output. The soma works as *the second layer*, which sums output from all the dendritic branches and produces the digitized output. There are several other studies in which a single neuron with active dendrites is regarded as a two- or multi-layered processor [19]–[24], like the model of Poirazi et al. (2003).

When a single neuron can work as a multi-layered processor, what elaborate function can we expect if such neurons are networked? Several authors have explored possible function exerted by a network of neurons with dendritic degrees of freedom [25]–[28]. Our study is in line with such exploration. We focus on the slow dendritic dynamics, called the dendritic plateau potential [6], [7], [9]–[11], [13]–[15], using the so-called neural field model [29], [30].

Among a variety of functions arising at the network level, the spatial working memory has been extensively studied without the role of active dendrites taken into account. Here we investigate the role of an active dendrite on localized activity, called a bump [30], [31] which has been considered to explain the spatial working memory. The spatial position of a bump in the network can be considered to represent the memory of a spatial position [32], [33]. A bump has also been proposed to be a mechanism for visual feature selectivity [34], [35] and a head direction control [36]–[38]. A bump may correspond to the localized cortical activity observed in various areas of the cortex [39]–[42]. The bump activity was extensively studied first with a rate model on a one-dimensional field model [29], [43]. It was later also studied with a spiking neuron network [44], [45]. With spiking of neuron was taken into account, the bump activity was shown to be intact as far as neurons within a bump fired a spike asynchronously. A bump was shown to be destabilized through partial synchronization. We discuss relations between their studies and the present study in Discussion. Laing and Longtin (2001) made an interesting contribution to the long-lasting debate whether or not a moving bump can be immobilized [46]. Using a one-dimensional simplified network model without dendritic dynamics, they showed that in vivo-like noisy input could immobilize a bump. The immobilized bump can be interpreted to memorize a particular position [32], [33]. However, the bump they examined was either permanently mobile or immobile for a given noise strength, and lacked flexibility. It would be even better if we could switch a bump between mobile and immobile modes as we like.

Here we consider a locally connected two-dimensional field model with active dendritic dynamics. Specifically, we simulated a two-dimensional network of 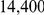 model neurons, each endowed with active dendritic dynamics imitating the dendritic plateau potential, localized large depolarization lasting tens or hundreds of milliseconds as observed in [6], [7], [9]–[11], [13]–[15]. The plateau potential is considered to be mediated by voltage-dependent 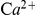 channels (VDCC) [7], [9] and/or N-methyl-D-aspartate (NMDA) receptor channels [6], [11], [14], [15]. The neurons were locally coupled in a manner observed in the cortical layer 2/3 pyramidal neurons [47], [48], and were intended to represent neurons in 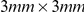 of the cortical area, which corresponds with the size of the rat primary visual cortex [49]. We find that only when the active dendrites have slow dynamics can a bump be both mobilized and immobilized in the same in vivo-like noisy condition. The coexistence of the mobile and immobile modes in the same condition shows clear contrast to the previous result [46] obtained without the dendritic dynamics taken into account. Importantly, we show that we can switch a bump from one mode to the other with a physiologically achievable transient signal.

The swift switching of a bump mode implies different functional advantages, depending on the cortical areas. In the visual cortex, a bump at a particular cortical position is considered to bias the information flow from a particular retinotopically corresponding position in the visual field (see [50]). A moving bump, thus, performs a visual exploration to locate any interesting or dangerous objects worth attending to. If an object of interest is found, a bump can switch to the immobile mode to keep attending to the particular object in the visual field. In an area responsible for memory storage, a mobile mode can be useful as a memory searching tool. Once the memory being sought is found, the bump switches to an immobile bump that corresponds to the retrieved memory.

First, we specify a computational model for our simulations and show that our network exhibits a bump solution consistent with previous studies. We then show that a bump is effectively immobilized by noise. The mechanisms of mobilized and immobilized bumps are discussed. Next, the coexistence of the mobile and immobile modes and the switching between them in the in vivo-like noisy condition are demonstrated. The last two sections are devoted to discussion and a detailed description of our methods.

## Results

### Two-field model using Izhikevich and Morris-Lecar dynamics

**Figure 1:**
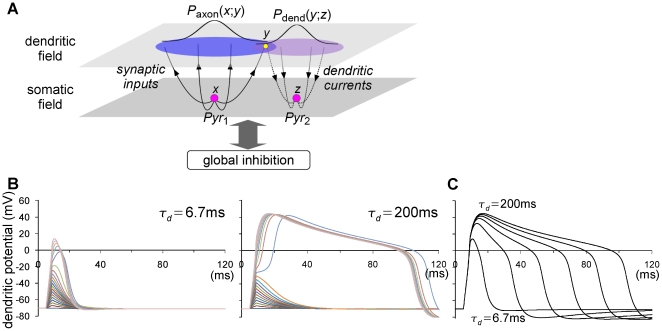
Two-field model. (A) A schematic introduction to the two-field model. The somatic (lower) and dendritic (upper) fields consist of two-dimensional arrays of somatic and dendritic units, respectively. The numbers of units in somatic and dendritic fields are 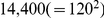 and 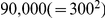, respectively. A somatic unit of the pyramidal neuron 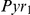 that is located at 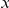 (left magenta dot), extends the axonal arbors to a dendritic unit that is located at 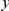 (yellow dot) according to probability 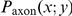. A somatic unit of the pyramidal neuron 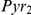 that is located at 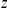 (right magenta dot), extends the dendritic branches to a dendritic unit that is located at 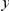 according to probability 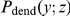. There are no direct connections between the units within each field. Global feedback inhibitory inputs are assumed. (B) Trajectories of the dendritic membrane potential with small 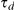 (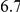 ms ; left) and large 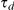 (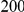 ms ; right). Current pulses, of which the intensity ranges between 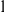 and 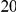 pA, were injected for 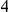 ms. Abrupt jumps in the amplitudes were observed. (C) The trajectory of the dendritic membrane potential changes depending on the dendritic time constant, 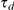, of which the values are 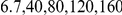 and 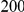 ms (lower to upper). The intensity and the duration of stimulation current pulses were 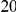 pA and 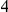 ms, respectively.

Our network represents a population of cortical pyramidal neurons with their massive intralaminar connections (Figure 1A). The pyramidal neuron, 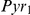, of which the soma is at 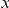 (2-dim position), extends its axon to 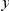 with probability, 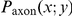. Another pyramidal neuron, 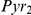, of which the soma is at 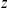, has its dendritic branch at 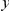 with probability, 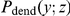. Thus, 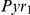 is connected to 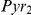 via a synapse at 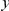 with probability, 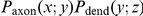. These connection probabilities decrease with the two-point distance. We describe this here with the Gaussian function to mimic observed connectivity between pyramidal neurons in the cortex, layer 2/3 [47], [48]. The whole network consists of a two-dimensional array of somatic units and a two-dimensional array of dendritic units, which in this paper we call the somatic field and dendritic field, respectively. We have 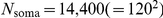 (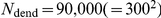) somatic (dendritic) units on the somatic (dendritic) field. At a given position on the dendritic field, multiple neurons' somata have their dendritic branches in common. According to the strategy of field modeling [29], those dendritic branches belonging to different neurons but occupying the same spatial position are assumed to have the same activation level and are represented collectively by a single dendritic unit. This field-model assumption is considered to work as long as the synaptic inputs are approximately continuous in space and the position is the principal determinant of the activation level. The signal transduction in our model is thus completed in two steps: a spike generated in a somatic unit at 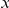 elicits a local excitatory postsynaptic potential (EPSP) in a dendritic unit at 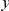, then the local EPSP propagates to a somatic unit at 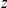 to elicit a somatic EPSP there.

To describe the dynamics of the somatic membrane potential, we employ the model proposed by Izhikevich [51], which involves an auxiliary variable to account for the effect of adaptation. To describe the dynamics of the local dendritic potential, we employ the Morris-Lecar model [52], which nicely elicits the voltage trajectories of the experimentally observed dendritic plateau [53] (Figure 1B and C). The time constant, 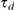, of this dynamics is the principal determinant of the duration of the dendritic plateau. The duration is not, however, equal to 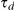. To clarify the role of the dendritic plateau in our simulations shown later, we used values of 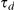 ranging from 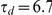 ms to 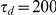 ms. A clear effect of the dendritic plateau potential is expected for large values of 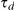.

In addition to the excitatory synaptic drive explained above, we need an inhibitory synaptic drive to prevent the neural activity from unlimited growth. Here, we used a global inhibition scheme in which one representative inhibitory neuron driven by the total activity of the network gave feedback inhibitory input globally to the all of the excitatory neurons. Details of our modeling are explained in Methods.

### Bump activity arises and moves on the network with noiseless background input

In the conventional field model based on point neurons, localized activity called a bump arises spontaneously with the architecture of the local excitation and widespread inhibition [29], [31]. Such bump activity is known to move in the noiseless condition as far as the dynamics involves some form of delayed adaptation or refractoriness [46], which typically exists in biologically realistic neuron models. Here, let us outline how a bump keeps wandering with a self-regenerative mechanism. Detailed explanations will be given later. We suppose here that a bump starts to move in a certain direction. Then the area trailed by the moving bump is in the middle of the refractory period, while the area ahead of the moving bump is yet to be in the refractory period. The refractoriness keeps trimming the trailing edge of the bump, while at the heading edge the bump keeps expanding. As a result, the bump continues to move in the direction which it has been moving. In other words, a moving bump keeps a gradient of refractoriness along the moving axis, which in turn keeps the bump moving, forming a self-regenerative loop.

**Figure 2:**
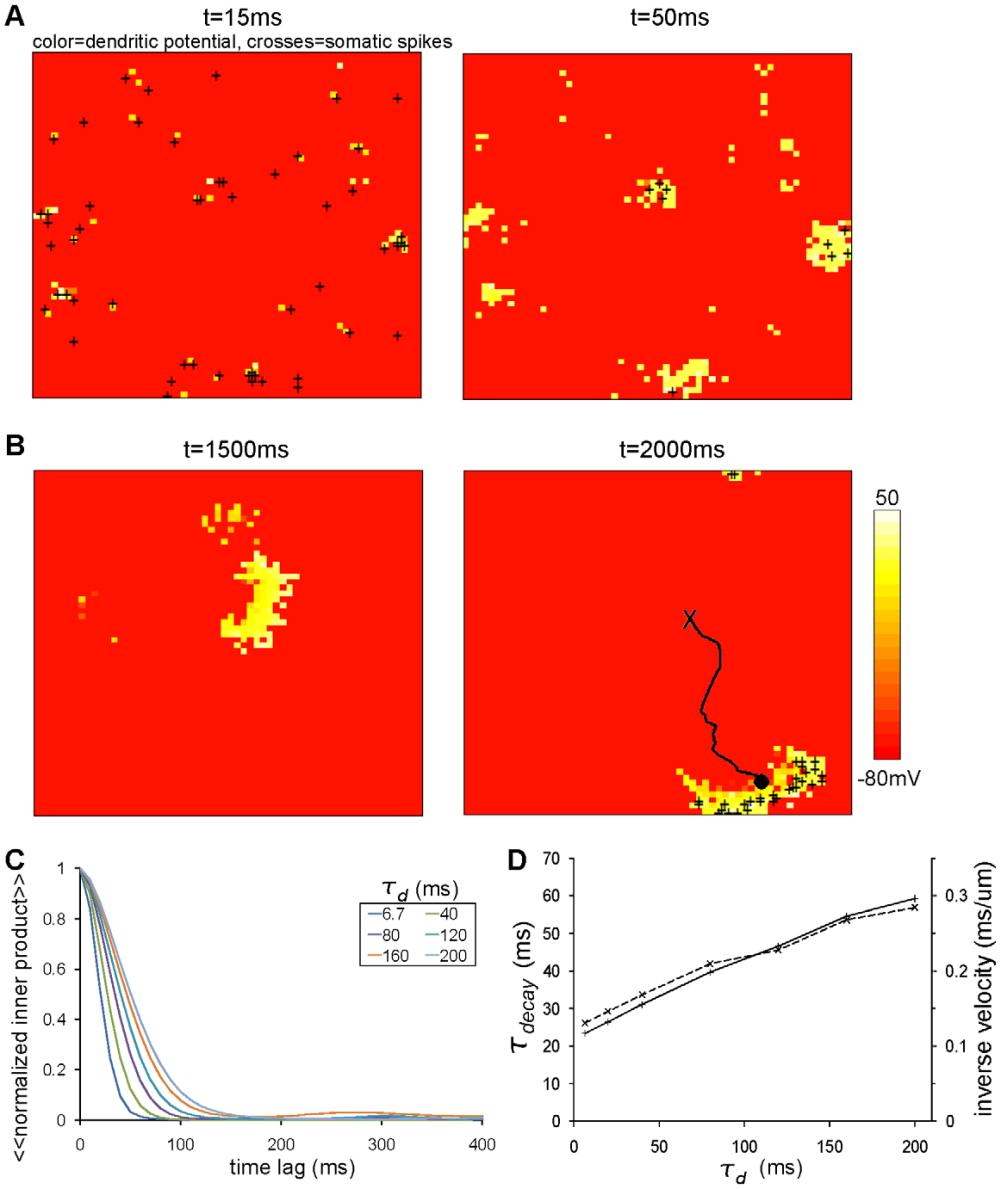
Spontaneous arising and moving of bump activity with noiseless background input. (A and B) The activity of the two-field model with noiseless background input (noiseless condition). Each panel is an overlaid view of the activity of somatic and dendritic fields. Heat-color at each location represents the value of the dendritic potential of the dendritic unit sitting at the corresponding location. The crosses indicate where somatic spikes occur at that time. Throughout the paper, panels of this type always represent 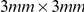 of the dendritic/somatic fields. The dendritic time constant was 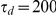 ms. The intensity of the noiseless background current was set to 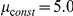 pA. (A) Spontaneously arising of small bumps. First at 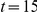 ms the neural activity was sparse (left), then the neural activity was spontaneously clustered and some small bumps were born by 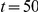 ms (right), which were later combined into a sizable bump by 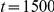 ms as in (B). (B) Spontaneous moving of a bump. The bump sitting near the center of the field (left panel) moves to the lower right (right panel). In the right panel, ‘X’ marks the center of the bump shown in the left panel, the black dot marks the center of the bump at 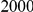 ms, and black line represents the trajectory of the bump. No crosses appeared at 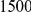 ms accidentally. (C) The inner product of somatic activity patterns showing rapid decay with a time lag for all of the values of 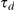. The curves were obtained by temporal and trial average of the inner products (denoted by 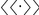). (D) The decay constant increased almost linearly with 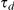. The decay constant was obtained from exponential fitting of the inner products in C (solid line). The overlaid dashed line indicates the inverse of the velocity of the center-of-gravity of the bump.

This self-regenerative loop, originally proposed for the mechanism of moving bumps in networks of point neurons, works for our network involving dendritic dynamics. Our simulations showed that a bump was spontaneously born (Figure 2A) and moved around (Figure 2B) in the noiseless condition, in which the constant and uniform background inputs were given to all of the somata during the simulation.

We first examine how the time scale of the dendritic dynamics, 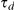, affects the moving bump. For simulations with all the values of 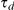 tested, a bump arises and moves. To measure the speed of the moving bump, a simple way is to measure the speed of its center of gravity, as shown by the dashed line in (Figure 2D). However, there are cases where a bump splits into two or more. Therefore, in the present paper we use a more robust way of estimating the speed of a moving bump, which is based on the similarity between the spatial pattern of activity at time 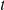 and that at time 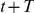. We measure the similarity as the inner product between the spatial patterns. The inner product depends on the time lag, 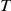. We therefore call it a time lag-dependent inner product, or simply inner product, of which the decay with the time lag reflects the movement of a bump in some cases including the present case, or the destruction of a bump in other cases. The inner product is always normalized such that the difference between the peak value at the zero time lag and the baseline is unity. For more detail, see Methods.

The inner product calculated from the simulations in the noiseless condition reveals a speedy and monotonic loss of the pattern of network activity (Figure 2C). The decay time constant, 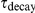, of the inner product determined by the single-exponential fitting gives a good measure of the speed of a bump. Indeed, the result of this is consistent with the result obtained by the speed measured with the center-of-gravity of a bump (Figure 2D). We found that as 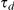 increases, 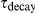 increases only linearly in a noiseless case (Figure 2D). Therefore, even with the slowest dendritic dynamics (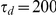 ms), the original position of the bump is forgotten rapidly, in a matter of several tens of milliseconds.

The quantification of the immobility of the network activity with the inner product is also good in its wide applicability. Although in the present paper, we are focusing mainly on bump activity, more distributed types of network activity can also convey some significant information. The decay time constant of the inner product quantifies the degree of information retention for such cases too.

### Dendritic plateau enables a bump to be immobilized with noisy background

**Figure 3:**
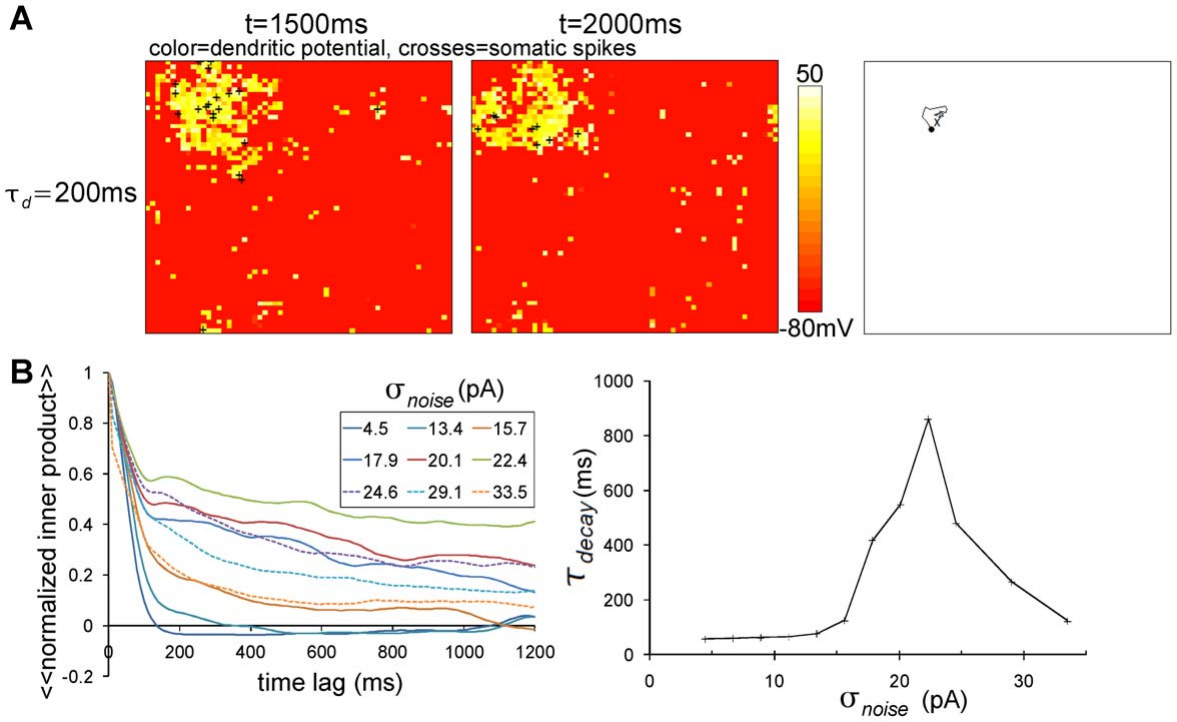
Bump remains immobile in noisy background condition with slow dendritic dynamics. (A) Snapshots at 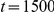 ms (leftmost) and 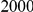 ms (center) and the trajectory of bump from 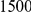 ms to 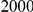 ms (rightmost) in noisy background inputs (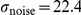 pA) with slow dendritic dynamics (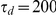 ms). The bump is practically immobile. (B) The decay of the inner product with time lag depends on the noise intensity, 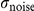. The left panel shows the decay curves of the inner product with a time lag for various values of 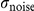. The curves were obtained by temporal and trial average of the time lag-dependent inner products expressed by 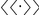. The right panel shows the dependence of the decay time constant calculated by curves of the inner product on 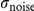. 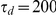 ms.

When the background input to the network was not a constant but fluctuating current or noise, as in the cortex in vivo, Figure 3A shows that the situation changed dramatically. With the long-lasting dendritic plateau (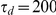 ms), a bump was spontaneously born as in the noiseless condition, but the bump no longer moved around rapidly. It stayed in the same position for at least 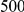 ms. In fact, the immobility of the bump increased nonlinearly with the level of the noise, as we can see in Figure 3B (left) that the decay of the inner product gets abruptly slowed at around 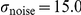 pA. Correspondingly, 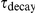 in Figure 3B (right) jumped up. It then went down with too strong a level of noise. Note that 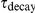 reflects not only the slowness of the bump motion, but also the stability of the bump existence against destruction by noise, then 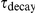 totally quantifies the invariability of the bump. Therefore, the observed decrease of 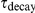 with too strong noise (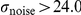 pA) means that the noise destroys structured activity, whereas the observed increase of 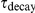 with moderately strong noise (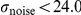 pA) means that the noise immobilizes a bump. The immobilization of the bump with the noisy background sounds similar to what was found in Laing and Longtin (2001) [46]. Our result is different however, in that a bump in our network can exist both in immobile and mobile modes under the same noisy condition, as we will see later.

**Figure 4:**
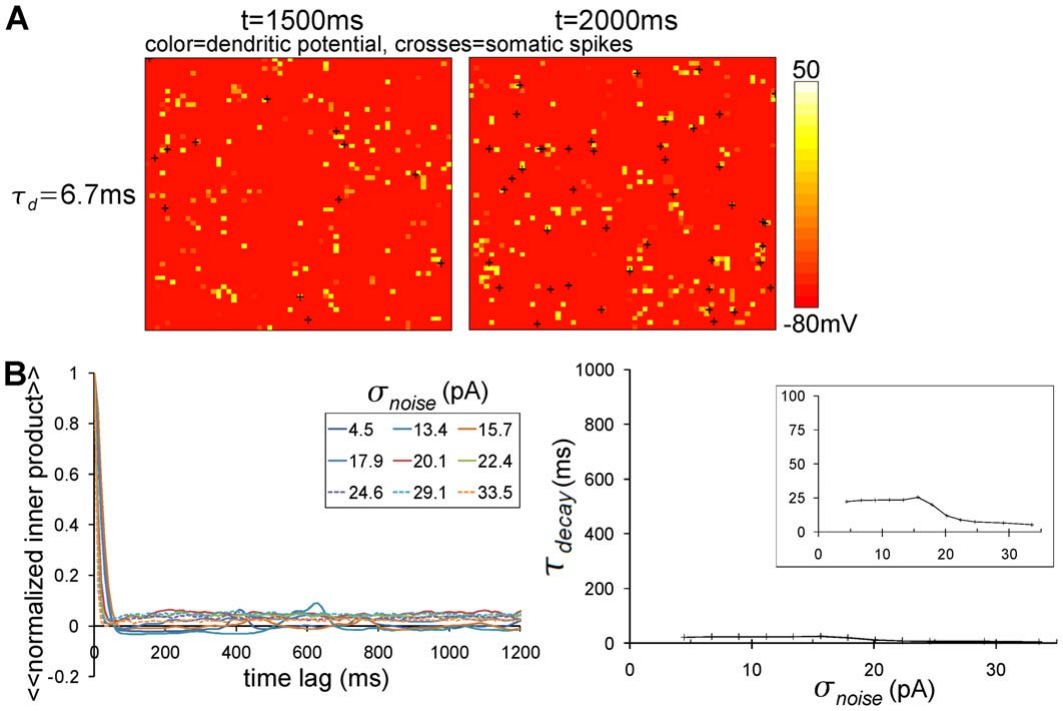
Bump is broken in noisy background condition without slow dendritic dynamics. (A) Snapshots at 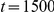 ms (leftmost) and 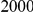 ms (center) of noisy background inputs (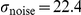 pA) with fast dendritic dynamics (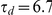 ms). No bump was observed. (B) The decay of the inner product with the time lag depends on the noise intensity, 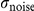. The left panel shows the decay curves of the inner product with the time lag for various values of 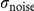. The curves presented are obtained with the temporal and trial average of the time lag-dependent inner products expressed by 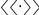. The right panel shows the dependence of the decay time constant calculated by curves of the inner product on 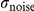. Inset: a close-up view. In this case, the inner products decay very rapidly in large values of 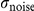 and any immobilization is not observed. 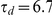 ms.

With the small size of the dendritic plateau (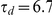 ms), the noise never resulted in an immobilized bump. The noise just broke up the bump (Figure 4). In other words, the long-lasting dendritic plateau protects a bump from breaking up and enables us to see the noise-induced immobilization. To see how this protection effect depends on 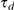, we plot in Figure S1B 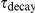 versus 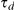 for 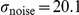 pA. We note that small values of 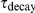 (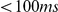) under this noisy condition mean that a bump is broken apart as in Figure 4B, while the large values (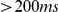) mean that a bump exists as in Figure 3A. Note that a plot of the same quantities, 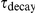 versus 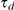, under the noiseless condition (Figure 2D) indicated a slowdown of a bump speed because a bump was always formed there.

Fitting of the curve of the inner product versus time with a single exponential function does not look reasonable in some cases (e.g. Figure 3B). We therefore tried the quantification of the decaying speed with more intuitive measure, the time by which the 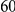
% reduction of the initial value of the inner products occurs (Figure S2). We found that the results were consistent with what were found with the exponential fitting.

**Figure 5:**
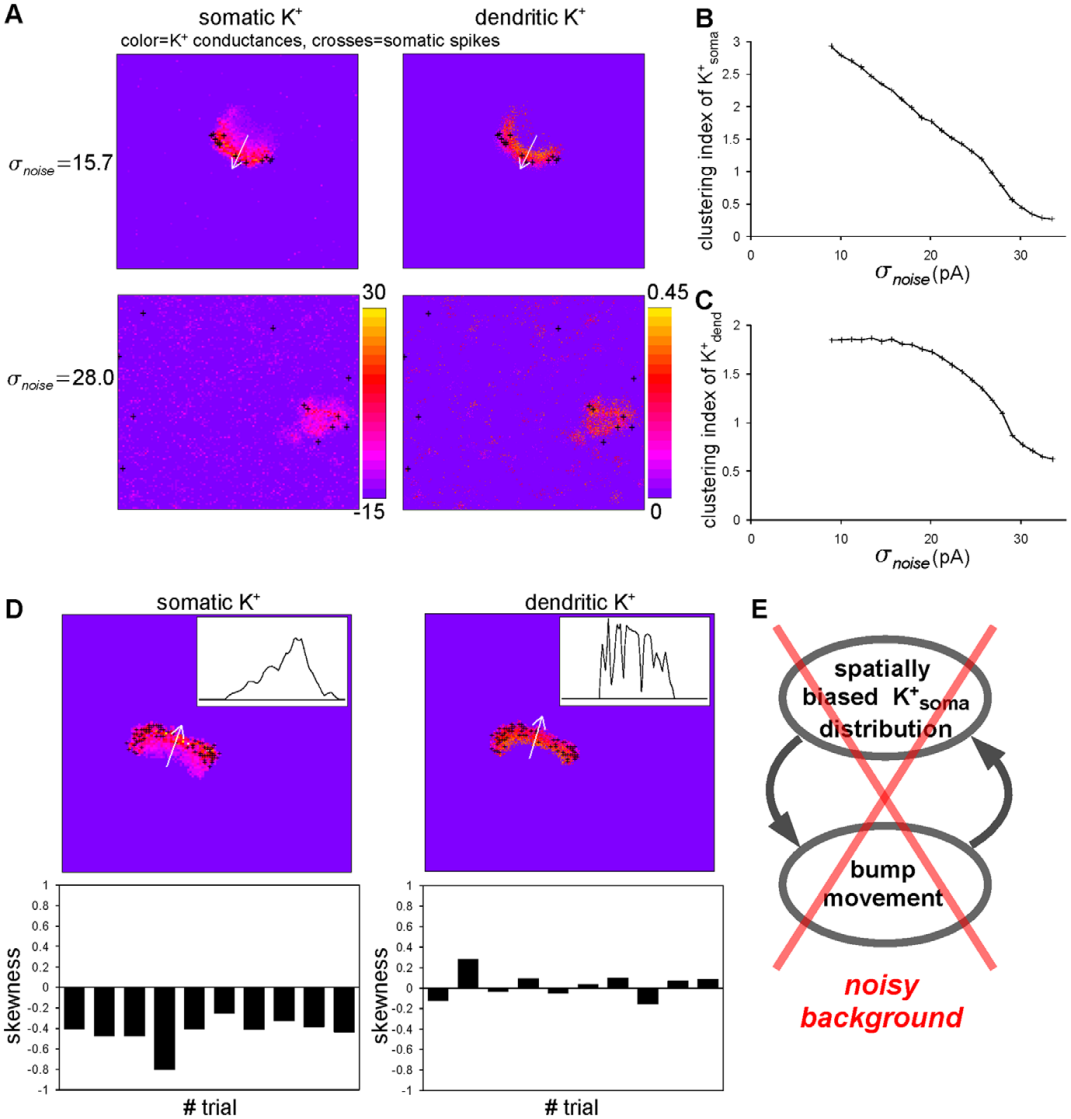
Mechanism for the moving and immobilization of a bump. (A) Each panel color-codes somatic (left) and dendritic (right) 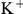 currents. Crosses depicting somatic spikes are overlaid on each panel. The 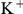 currents were 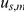 and 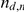 described in Methods. The crosses indicate where somatic spikes occur at the present time. The dendritic time constant was set to 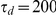 ms. In a weakly noisy condition (upper row; 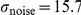 pA), somatic 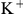 current is distributed in a spatially biased manner with its long-tail (left), whereas dendritic 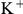 current is distributed in a relatively unbiased manner without a clear tail (right). In a strongly noisy condition (lower row; 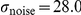 pA), the bump is widely spread and 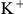 currents appear ubiquitously on the field. (B and C) Spatial clustering of 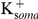 and 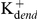 depends on noise intensities, 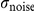. Both activities of 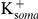 (B) and 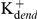 (C) are clustered in weakly noisy background inputs, and become distributed with an increase in the noise intensity. (D) Spatially biased distribution of somatic 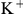 activity (left) and unbiased distribution of dendritic 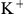 (right) activity along the direction of the bump movement indicated by the white arrow in a weakly noisy background. 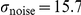 pA. The inset shows the one dimensional distributions of the 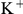 current by the white arrows. The skewness values calculated from the one dimensional distributions are shown at ten different points in time. In somatic 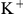 activity, spatially biased distributions are clearly found. (E) A schematic diagram showing the self-regenerative loop. In the noisy condition, the bump is immobile because generation of the loop is obstructed by the noise.

**Figure 6:**
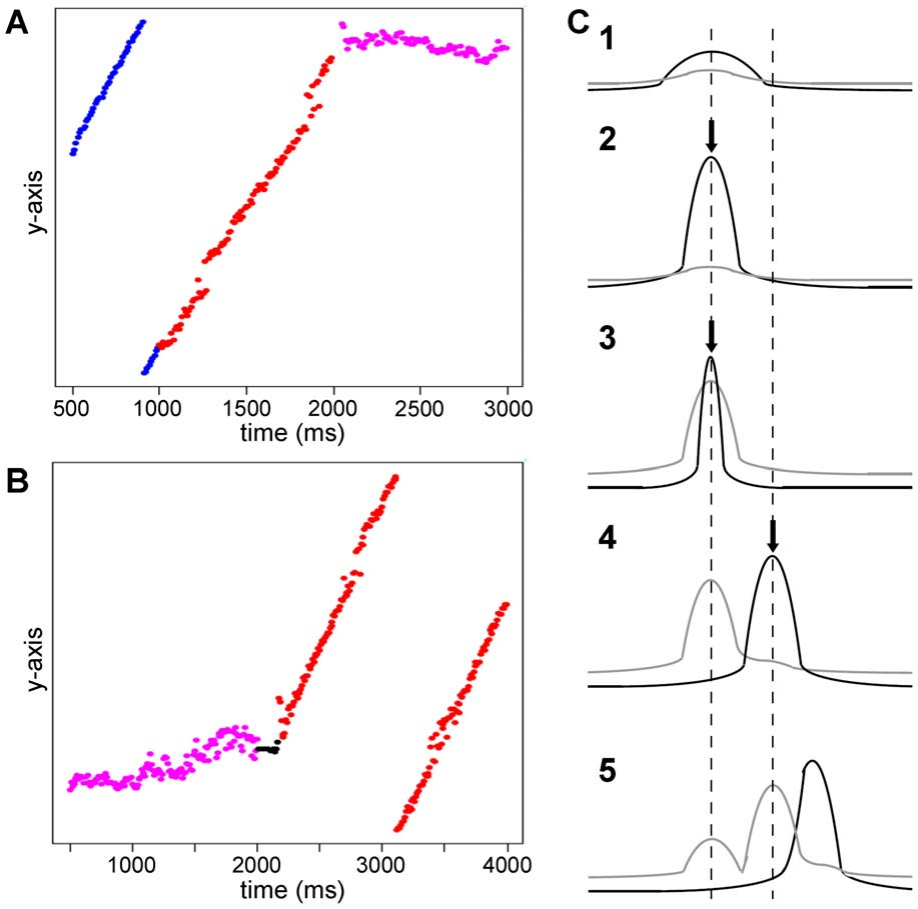
Bidirectional switching of bump mode in the noisy background condition. (A, B) The positions of the bumps in a linear strip (periodic boundary condition) are plotted versus time. Constant, 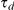, was set to 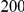 ms. (A) A simulation run started out in the noiseless background condition and remained in the condition until 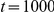 ms (blue dots), and the noisy background condition (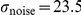 pA) started and continued until 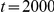 ms (red dots). Finally, a transient strong inhibition was applied globally from 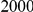 ms to 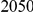 ms, and the bump was immobilized (magenta dots). (B) A simulation run started out in the noisy background condition (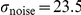 pA) (magenta dots), and at 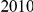 ms, a transient strong excitatory input lasting 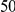 ms was applied on a thin vertical line at the center of the bump. Immediately following the excitatory input, another excitatory input was applied for 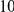 ms on a thin vertical line adjacent to the previously applied position. This combined excitation (black dots) switched a bump mode from an immobile one (magenta dots) to a moving one (red dots). (C) Schematic figure explaining how an immobile bump starts moving after the combined excitation is applied. Each panel from C1 to C5 show each stage of a process of starting for the bump to move. Somatic activity is depicted with the black curve. The influence of inhibitory effects due to somatic 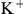 currents and the feedback inhibitory synaptic currents collectively is depicted with the gray curve. Arrows indicate the external application of the first (in C2 and 3) and the second (in C4) excitatory inputs. For more detail, see main text.

**Figure 7:**
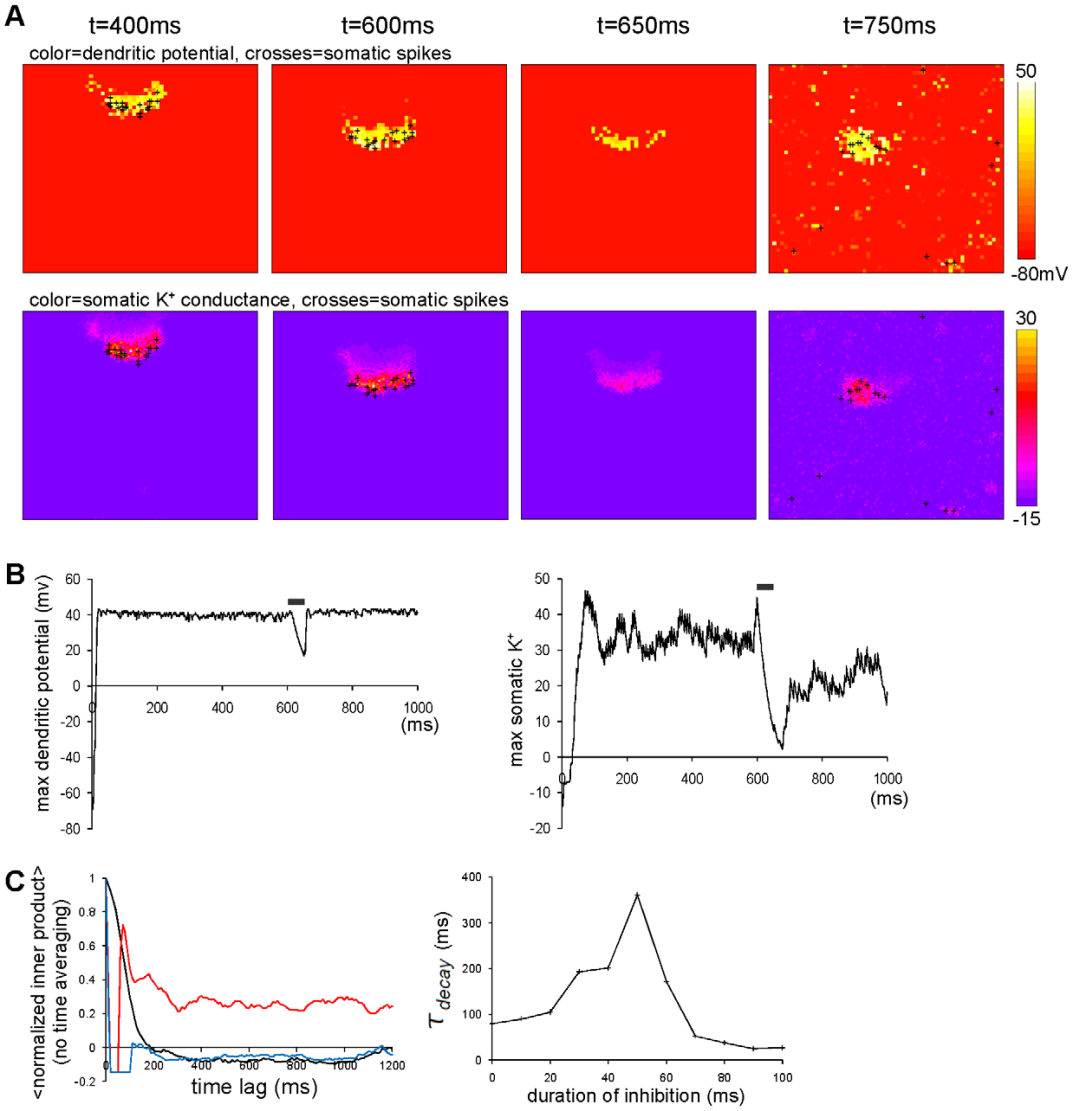
Determinant of inhibitory period for switching a bump from moving to immobile modes. The simulation runs where background inputs changed from noiseless to noisy currents via the varying length of the inhibitory period. The noiseless current is defined solely by the constant part 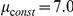 pA, while the noisy current is defined solely by the noisy part 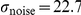 pA. 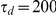 ms. (A) Snapshots for dendritic potential (upper) and somatic 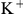 activity (lower) in the noiseless condition (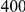 ms), at the switching point between the noiseless condition and inhibition period (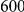 ms), at the switching point between the inhibition period and the noisy condition (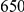 ms), and in the noisy condition (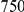 ms). The meaning of the symbol and color are same as the previous figures. (B) The time courses of maximum values of the dendritic potential (left) and the somatic 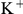 activity (right) in the simulation run the same as (A). The gray bar indicates the inhibition period. Whereas the somatic 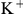 activity substantially drops, the dendritic potential and 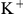 activity are kept at large values. (C) (left) The inner products between the activity at the switching point from the noiseless to inhibitory period (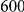 ms) and the subsequent activities. The durations of inhibitory periods are 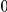 ms (black), 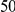 ms (red) and 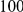 ms (blue). The rapid decline in the black line means that the change from the noiseless to noisy condition without an inhibitory period did not immobilize the bump. The high values maintained in the red line mean that the inhibitory period successfully immobilized the bump, which did not work well when the inhibitory period was too long (blue). Note that each of the curves were obtained by the trial, but not the temporal average of the time lag-dependent inner products, which are denoted by 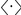. (right) Dependence of the decay time constant of the inner product on the duration of the inhibitory period.

**Figure 8:**
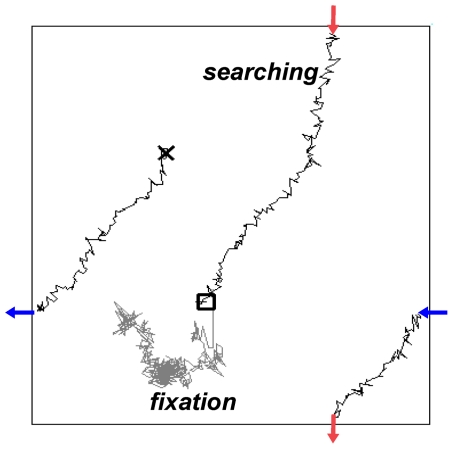
Searching and fixation by the present mechanism. First, a bump moves on the cortical field under noisy condition (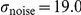 pA). Its two-dimensional trajectory is indicated by the black line starting at ‘X’. This movement corresponds to the search in the retinotopically corresponding visual field if the visual cortex is considered. When something worth attending to (of which the retinal image is in the black square) is found, the transient global inhibition whose duration is 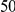 ms as in Figure 6A, immobilizes the bump around the region of interest, which corresponds to the fixation of visual attention. The two-dimensional trajectory after this transient input for 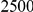 ms shows its immobile nature (gray line). Note that the trajectories going outside from one sides of the field expressed by the outward arrows come in at the other sides expressed by the inward arrows of same color because of periodic boundary condition. In this simulation, 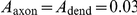, 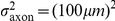, 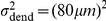.

A technical note: in the forthcoming Figures 5, 6, 7 and 8 we used a smaller and supposedly more realistic value of EPSP compared to that used in Figures 2, 3 and 4, which was necessary for comparison among diverse values of the dendritic time constant, 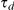. We confirmed that the use of smaller EPSP size did not affect the important observations (see *Simplifying assumption* in Discussion and Figure S3AB).

### Mechanism

In this section, we consider how the noise-induced immobilization happens. On a network of point neurons involving delayed refractoriness, bump activity is known to move around in a noiseless condition [46]. We first examined whether the same mechanism worked in our model involving dendritic dynamics. In our model, both somatic and dendritic membrane potential dynamics involve the delayed refractory mechanism. These are mediated respectively by currents 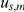 of the somatic dynamics (Eqs. (1–2)) and 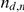 of the dendritic dynamics (Eqs. (3–7))(see Methods). Since these currents play functionally the same refractory role as the potassium currents in real neurons, in this paper we call these currents somatic and dendritic 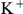 currents.

If we consider a moving axis (white arrow) of the bump in Figure 5A, upper, somatic spikes (black crosses) are concentrated at some point on the axis. In reference to the spiking position, the somatic 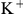 currents are distributed in a spatially biased manner along the axis, while the dendritic 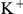 currents are distributed in an almost spatially unbiased manner. Such spatially biased (unbiased) somatic (dendritic) 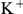 currents are illustrated more clearly in Figure 5D, where the current distributions along the moving axis are depicted. The somatic spikes in the bump in Figure 5A, upper, deliver driving currents isotropically due to its symmetric innervation (see Figure 1A). However, the currents delivered in the tail direction cannot elicit spikes because the long tail of the somatic 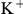 current distribution inhibits it. On the other hand, the currents delivered in the head direction can easily elicit new somatic spikes because there is a fresh field free from refractory currents at the head. Thus elicited new spikes drive the bump in the direction in which it has been moving. Importantly, the movement keeps the somatic 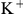 current distribution spatially biased, while the spatial bias keeps the bump moving, resulting in a self-regenerative loop (Figure 5E). Thus, once it has started moving, the bump keeps moving. This observation corresponds well to what happens in the point neuron model [46], and in this bump movement, the delayed refractoriness of somatic but not dendritic 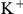 currents plays a major role.

In contrast, strong noise (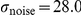 pA) was able to disrupt the generation of the self-regenerative loop (Figure 5A, lower). Strong noise elicited spontaneous firing everywhere, leading to after-spike 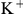 currents appearing ubiquitously on the field, as shown in Figure 5A, lower, and also in Figure 5B and C, where the magnitudes of spatial ubiquitousness of 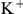 currents are quantified for varying strengths of background noise. In such a situation, somatic 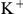 current no longer showed a clear spatial bias of distribution. The bump, therefore, had no tendency of moving to a particular direction. Therefore, the bump did not ignite the self-regenerative loop. Taken together, a bump could keep moving if it was born and existed in a noiseless clean environment, while a bump remained immobile if it was born and existed in a noisy environment.

Next we examined what would happen if a bump was born in a noiseless background and started moving, and the background input current was later changed to a noisy one. Actually, the bump could not be immobilized after it had already been engaged in the regenerative loop, as illustrated by the blue (noiseless background) and red (noisy background) dots in Figure 6A. The movement of a bump is restricted within a linear strip in the simulation run here, so that the bump position is quantified with a single coordinate. We confirmed in simulation runs without restriction that the same phenomenon (failure of immobilization) happened (see Figure 7C, black line). In Figure 6A, the linear part in 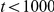 ms represents the bump movement in the noiseless environment and the subsequent linear part in 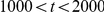 ms represents the bump movement unaffected by the noise added later. Generally, strong noise elicits the somatic 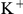 currents everywhere, as we see in Figure 5A (lower panels), which can potentially impede and slow down a moving bump. But even the strong noise cannot actually immobilize the already moving bump because the self-degenerative loop is robust. If we increased the noise level further, the bump was broken up. These observations mean that a bump is bistable in a noisy background. Depending on the history, a bump can be either mobile or immobile in the same noisy condition. Such robust bump movement persisting in noise is also demonstrated in the movie, Video S1. The bump could finally be immobilized later at 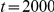 ms with a protocol explained in the next subsection.

### Switching the bump mode

The simple addition of noise was shown not to immobilize an already moving bump, as we see in the simulations with (Fig. 6A) and without (Fig. 7C) restriction of the bump movement to a linear domain. We were curious about how we could convert a bump from mobile to immobile modes. To immobilize a moving bump, we found that we needed first to apply a transient (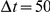 ms) strong global inhibition to the somata before starting the noisy current injection. The duration of inhibition was meant to match the decaying time constant of the somatic 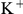 current, 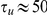 ms. Figure 7A illustrates how a moving bump actually reacted to this combined stimulus, and Figure 7B illustrates the time courses of maximum dendritic potential and maximum somatic 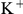 conductance over the field for each instant of time. In this simulation run, the bump was born and was moving in the noiseless background until 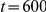 ms. The inhibitory input starting at 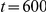 ms suppressed the somatic spikes, reducing the somatic 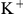 currents as shown in Figure 7A (around 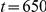 ms). By the time the transient inhibition ended, somatic 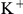 conductance had been so largely lost (see Figure 7B, right) that somatic 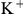 could no longer determine where new spikes should preferably occur. Therefore, the soma started firing in an unbiased manner, as illustrated in Figure 7A (upper row at 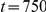 ms). The bump thus “forgot” the direction in which it had been moving. However, the bump still “remembered” where it had been located, as the dendritic potential still kept its activity because of its slow dynamics (see Figure 7B). Thus, the moving bump was immobilized at the location where the bump was just before starting the inhibition. For this reason, the separation of time scales between dendritic dynamics and somatic 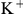 dynamics (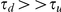) is essential for the conversion.

In this condition, the inhibition period 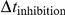 must be comparable to or longer than the time scale of the somatic 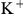 and be shorter than the time scale of the dendritic dynamics (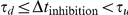). The most effective duration transpired to be 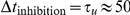 ms (Figure 7C, left, red line) in our simulation setting, as shown in Figure 7C, right. As we mentioned above, the simple addition of the noisy background inputs did not effectively immobilize the bump (Figure 7C, left, black line), while too long an inhibition erased locational information of the bump (Figure 7C, left, blue line).

To clearly illustrate such noise-induced immobilization assisted by the inhibition, again we ran the simulation where the bump movement was restricted to a linear domain, as in the last part of the last subsection. As shown in Figure 6A and Video S1, the bump switched from mobile to immobile modes around 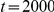 ms when the inhibition was input. Note that in this simulation, the inhibitory period was sandwiched by the same noisy background periods, expressed by red and magenta dots in Figure 6A.

The inhibitory input was given to the somata here because in the living brain, the inhibition is largely provided by the basket cells, which are known to target mainly soma or proximal dendrites as the switching stimulus used above [54].

Next we tried to find a protocol for the reverse conversion: from an immobile mode to a mobile mode in a noisy background. After our search, we found that a transient excitatory input (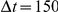 ms) on the immobile bump followed by a flash (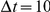 ms) of another excitatory input to the position adjacent to the initial excited zone ignited the bump movement (Figure 6B and Video S2). This combination of inputs can cause the spatially biased 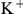 current distribution that drives the bump movement, which is shown schematically in Figure 6C. First, the bump is immobile (Figure 6C1). Upon the external application of the first excitatory input, the activity level increases while the inhibitory effect is yet to increase because it takes some time for the inhibitory effects to go up (Figure 6C2). The inhibitory effects go up later (Figure 6C3). When the first excitatory input has finished and the second excitatory input at the adjacent position is given, the activity level at the new position goes up and that at the old position is lost, which shifts the position of bump activity to the right. However, the inhibitory effect at around the old position still remains because of the slow dynamics of the 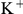 current (Figure 6C4). A little later, the slow 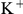 current seen in (Figure 6C4) is a little decayed but still remains in (Figure 6C5), and combined with the additional 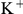 current resulting from the bump activity in (Figure 6C4) (the higher peak) causes a spatially biased distribution of the 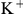 current, which means that the rightward movement continues.

In summary, we found that we were able to convert a moving bump to an immobile bump by transient (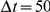 ms) global inhibition, and were able to convert an immobile bump to a moving bump by a transient (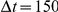 ms) targeted excitation followed by the flash (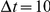 ms) of another targeted excitation in noisy background inputs.

## Discussion

In the present paper, we have studied the effect of the dendritic plateau on network dynamics. We first confirmed in the noiseless condition that the localized activity of a bump arose spontaneously and moved on our network model involving dendritic dynamics. With the noise added, an unexpected observation was made for a model with slow dendritic dynamics. In a model with slow dendritic dynamics (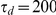 ms), a bump that moved around for smaller noise levels no longer moved around for larger noise levels. For still greater noise levels, the bump just broke up. Therefore, there exists an appropriate noise level at which a bump is immobile. This mechanism did not work for a model without slow dendritic dynamics. In a model with fast dendritic dynamics (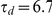 ms), for example, a bump broke up before it could possibly be immobilized by the noise. Interestingly, we could also observe a moving bump with the dendritic time constant, 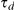, and noise level, 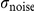, with which we observed a immobile bump, meaning the presence of the coexistence of immobile and mobile modes. Moreover, a bump could switch between mobile and immobile modes with physiologically feasible stimuli. How such switching stimulation actually occurs in the brain is among our important future works. The coexistence of the two modes distinguishes our study from the previous study [46], which demonstrated a moving bump in the noiseless condition and an immobile bump in the noisy condition, but not both modes coexisting in the same condition. Our new observations represent an interesting, unexpected role played by the slow dendritic dynamics in the network activity of neurons. We give a table summarizing our observations in Table S1.

Among a family of models with varying values of 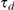, a particular version specified by the fastest dendritic dynamics (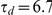 ms) is considered closest to the model of [46], which we call a point-like version. With the point-like version, we could not immobilize a bump. Meanwhile Laing and Longtin (2001) [46] did find an appropriate noise level for immobilization with one but not all of the models they studied. We therefore consider that whether a network of neurons with fast (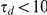 ms) dendritic dynamics can immobilize a bump or not depends on fine details of the model specification. In contrast, a network of neurons with slow dendritic dynamics showed a robust immobilization under the noisy condition. From our repeated simulation runs with a varying level of noise, we could easily find a range of noise levels that immobilized a bump. We therefore consider that slow dendritic dynamics may have widened the parameter range of the noise intensity resulting in the bump immobilization.

### Functional significance

As a possible functional role of the bump activity, several different possibilities have been proposed to date [32]–[38], [43]. With our new observation of the coexistence of the two distinct bump modes, additional possibilities arise, as we describe below.

The dual mode in the bump activity is expected to play a functionally significant role, differently in different areas of the brain. The bump activity we observed in the modeled cortical network is analogous to what was found in the rat primary visual cortex (V1) with voltage sensitive dye [42]. The neurons constituting the bump activity are hyperactive, so they tend to respond to a visual stimulus more easily than the neurons outside the bump do. In this sense, the bump works as a gate that selectively lets through the visual information mapped to the position of the bump. Analogous gating of sensory information by the ongoing activity has been suggested by in vivo recordings from the visual cortex [50]. The whole 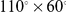 of the right visual field of rats is retinotopically mapped to the left V1 cortex [49], and a bump on the cortex corresponds to a particular position in the rats' visual field.

The main interest of carnivorous animals is to hunt others, and so they have a small but highly resolved visual field. In contrast, the main interest of herbivores and certain omnivorous animals, such as rats, is to provide the best risk surveillance by means of a less resolved but very wide visual field. With the wide visual field, they do not need to move their eyes as frequently as hunting animals whose high-acuity visual field is very small. Although there is little need of moving their eyes, we considered that they could and would still set the focus of attention by putting a bias to information coming from a particular part of their wide visual field. Such an attentional function with a *software* mechanism instead of a hardware mechanism like the saccade would be beneficial. Once rats find an object that is worth attending to for their safety or their diet, they need to fixate the point of attention. As we have demonstrated, a moving bump in our network can switch to the immobile mode with physiologically feasible stimulus, so that would provide a mechanism to fixate an important object in the wide area of their visual field (Figure 8 and Video S3).

If we consider that our model describes cortical or hippocampal areas where memory traces are stored in a spatially confined manner, we can regard the moving bump activity as an effortful or effortless search for memory, and regard the conversion to an immobile bump as memory recall. Specifically, we assume that memory traces are stored in different places within an area for storage of information, and that activation of a certain place implies the recall of the corresponding memory trace. On this assumption, a bump moving in a storage area is interpreted as a series of the retrieval of various memory traces. It is like browsing through stored information. With the mechanism proposed here, when the information sought is found, a bump is supposed to be immobilized at the corresponding position and the corresponding memory trace is kept activated.

Thus, two distinct kinds of function of the switchable bump can be considered. Although we considered Gaussian probabilistic connectivity between neurons in our simulations, we could also consider more structured connectivity to suppress or enhance stochasticity of bump movement and to give the network some functionality. The possible benefit of such connectivity is worth studying in the future.

### Related computational studies

A bump arising in a network model with local coupling has a long history of study dating back to [29], [43]. For its mathematical tractability and its functional interest concerning spatial working memory, there is a long list of interesting studies. Please refer to [30] and the references therein. Most of these studies work in a one-dimensional setting. Our study is new in that we have added active dendritic dynamics to this line of studies in a two-dimensional setting in the noisy condition. We then found that mobile and immobile modes coexist.

Our network is based on a spiking neuron model unlike many other network studies which based on a rate neuron model. Laing and Chow (2001) studied a bump solution. Their study was also based on a spiking neuron model [44]. It is therefore instructive to see similarities and differences between this study and the present study. Laing and Chow (2001) used the local inhibition to model inhibitory synapses and did not consider the adaptation currents or the dendritic effects, while we adopted the global inhibition and explicitly modeled the adaptation 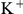 currents and the dendritic dynamics. They asked how the time constant of the synaptic current affect a bump movement and found that three different modes of a bump motion appear depending on the time constant: stationary, wandering and traveling bumps. When the synaptic transmission was slow, the bump did not move which was called ‘stationary’. As the synaptic time constant decreases, the bump changed from a stationary mode to a ‘traveling’ mode via ‘wandering’ mode characterized by a Brownian motion. They observed that the speed of a traveling bump became faster as the synaptic time constant decreases. Chow and Coombes (2006) studied these phenomena with a network of a simpler phase neuron model [45], giving an analytical insights. Despite structural differences between those models and the present model, we observed some similarities in a bump behavior. Specifically, our observation under the noiseless condition that the faster dendritic dynamics implied the faster movement of a bump (Figure 2D) is similar to the observation in those models that faster synaptic kinetics implied a faster bump movement if we equate the dendritic time scale and synaptic time scale. This similarity is however found limited if we compare the details. In fact, even at slowest time scale, our bump looked still ‘traveling’ and we never observed a ‘stationary’ or ‘wandering’ bump in the noiseless condition.

As for the types of a bump, it seems that our mobile bumps are categorized as a traveling bump. The movement of a bump we observed either under noisy condition or noiseless condition on the linear strip looked very much like constant-speed motion, and it appears to be a traveling bump as is seen in the simulations (Figure 6AB and Video S1 and S2) unless a bump was in the immobile mode (the pink trajectories in Figure 6AB). Such a bump seems to be a traveling bump also in that a bump was never observed to switch moving direction. Observed directional motions resulted from the adaptation 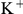 currents, suggested in Figure 5. Since exactly the same mechanism should work irrespective of the dimensionality of the field, we believe that bump motion on the non-restricted 2D field (Figure 2B) is also in a traveling mode. Because there are many more moving directions are allowed on the non-restricted 2D field than on the linear strip field, the bump in the non-restricted 2D field tends to be wiggling (Figure 2B). This larger freedom of motion together with stochasticity coming from the finite size effect and noise makes a bump motion look like wandering on the 2D field. However, the motion is not purely Brownian because there is always a prohibited direction, which makes our mobile bump different from the wandering one.

The immobile bump in our simulations (Figure 3A) is a single notable exception. We think that this bump is regarded as a wandering bump. First of all, there was no spatially biased distribution of adaptation currents observed for an immobile bump, so that the bump can not be a traveling one. Since the immobile bump in our model exists only under the noisy condition, it cannot be a stationary bump, either. Taken together, we think that our immobile bump appearing under the noisy condition with slow dendritic dynamics is regarded as a wandering bump and the mobile bumps are regarded as a traveling bump.

To shed light on the computational roles of the nonlinear dendritic dynamics, several theoretical studies have been performed at cellular or network levels. As mentioned in the Introduction, several authors have emphasized that active dendritic branches would enhance the computational power [17]–[24]. Larkum et al. (2004) focused on the computational role of layer 5 pyramidal neurons that extend their dendrites up to layer 1, the locus which the top down attention signals target [55]. They focused on the experimental observation that dendritic 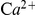 spikes in the distal apical dendrites activated by backpropagating action potentials result in gain modulation of the layer 5 pyramidal neurons. They argued that the observation implies an association between top-down and bottom-up inputs. Remme et al. (2009) showed that dendritic branches can be regarded as oscillators weakly coupled and phase-locked with each other [56]. Following this, Remme et al. (2010) showed that the phase-locking affects the dendritic independence and the interaction between dendritic oscillation and somatic activity, which disrupt grid field formation [57].

Functional implications of a network of neurons with active dendrites have been proposed by several authors, as referred to in Introduction [25]–[28]. Koulakov et al. (2002) and Goldman et al. (2003) studied the role of active dendrites on the so-called parametric working memory based on the neural integrator [25], [26]. The originally proposed mechanism requiring the fine tuning of parameters was replaced by a much more robust mechanism proposed by them, which was based on the bistability of the dendritic membrane potential caused by an active dendrite. Goldman et al. (2003) further showed that multiple bistability among different dendrites would reproduce an observed integration property. Morita (2008) pointed out that the local dendritic integration is beneficial in improving accuracy of the encoding stimulus because inevitable noise arising in every dendrite can be cleaned at each dendrite locally because of the non-linear dynamics and the local noise is prevented from spreading globally [27]. In contrast to our study focusing on a possible role of slow dendritic dynamics in the network activity, Memmesheimer (2010) studied a possible role of fast dendritic dynamics in the network activity [28]. His results indicated that each dendrite works as a coincidence detector. The coincidence detected in dendrites promotes somatic spikes with high temporal accuracy, leading to a highly synchronous network activity. Since such network activity is occasionally shut down by refractoriness or inhibitory inputs, he argued that this mechanism could explain the observed sharp wave ripple.

Koulakov et al. (2002) [25] and Goldman et al. (2003) [26] in particular studied the role of a bistable dendrite that was probably caused by NMDA conductance, which is the main factor of the dendritic plateau we incorporated into our present model, where the network structure is very different from theirs. As a consequence, we elucidated a different aspect of the functional role of NMDA conductance on the network dynamics. At a single-neuron level, a possible role of the dendritic bistability in the parametric working memory was discussed in [58].

Although the present study considers moving due to the delayed refractoriness or adaptation, a bump can move around even without such a mechanism due to heterogeneity in the network. Renart et al. (2003) studied the moving of a bump of this kind, and discussed the switching of a bump from mobile to immobile mode from a perspective different from ours [59]. They demonstrated the switching of a bump mode occurring due to synaptic plasticity. Therefore, the time scale of switching in their study is much longer, typically hours to days, than our case of 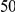 ms.

### Simplifying assumption

In our simulations that produced Figures 2, 3 and 4, we used an EPSP size larger than that commonly considered. We used such a size when and only when we needed to vary the size of 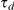, because with the large EPSP size, no parameter tuning accompanied by a change in the 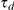 value is needed and make our compuation inexpensive. The large EPSP size turned out not to affect the important observations. In fact, our network simulations with the EPSP size 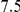 times smaller than that used in the previous one reproduced the noise-induced immobilization with the slow dendritic dynamics (Figure S3A and B).

We note that noise generally entails the risk of breaking up a bump before it immobilizes the bump. However in our modeling, the immobilizing effect (Figure S3A) turned out to be strong and the bump was immobilized without destruction, as is seen in the inset in Figure S3A. The destructive effect quantified in Figure S3C was mild. When we defined the destruction-corrected degree of immobilization (Figure S3D) as a product of Figure S3A and Figure S3C, it still showed a clear peak. Therefore we used the small EPSP size in Figures 5, 6, 7, 8.

### Consistency with physiologically known facts

For our proposed model, we made several assumptions which required careful consideration. First, we intended our network to represent the layer 2/3 cortical circuit. We assumed that neurons there were highly interconnected. This assumption is supported by the observation in [60], showing that excitatory interconnections in layer 2/3 are much richer than are both afferents to layer 2/3 and interconnections in other layers. Second, we assumed probabilistic connections among units described by the Gaussian function. Some anatomical and electrophysiological studies have implied a Gaussian connectivity between pyramidal neurons [47], [48], while other studies have implied a more structured connectivity [61]–[64]. Elucidating the possible consequences of such structured connections is an important future issue to be addressed. Third, we assumed that inhibitory inputs targeted only somata. We consider that this is a good first approximation because a basket cell, which is the principal inhibitory neuron in layer 2/3, sends 50% of its terminals to the somata or proximal dendrites [54]. Considering also inhibitory neurons that synapse on distal dendrites is, however, an interesting future direction. Fourth, we let a single inhibitory unit represent the population of inhibitory neurons by assuming that one unit received the total neural activity and sent its inhibitory drive back to the excitatory neurons. Therefore, the inhibitory unit is not a single inhibitory neuron. Although this assumption was meant to simplify the computational modeling, it may also have physiological relevance if we consider the observed gap junctional networks of inhibitory neurons [65]. It is still a matter of debate whether the gap junctional network extends only locally or globally, while an immunohistochemical study has suggested that the gap junctional network is boundless [66]. Instead of letting a single inhibitory unit represent the total effect, we can assume multiple inhibitory units exert an inhibitory drive. In this case, multiple bump solutions may be formed. This can then greatly enlarge the potential for information processing of a network of neurons with dendritic dynamics, and is an interesting possibility.

The final and most fundamental assumption of this study is the existence of the dendritic plateau potential in vivo. There are lots of in vitro reports of the dendritic plateau in the cortex and hippocampus [6], [7], [9]–[11], [13]–[15]. Nevertheless, direct evidence of it through in vivo observation remains to be found because of the experimental difficulty. Several lines of indirect evidence of other types of dendritic action potential in vivo exist. A combination of intracellular recordings and two-photon 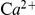 imaging has revealed spontaneous and sensory-evoked dendritic 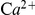 action potentials in apical distal dendrites of layer 5 pyramidal neurons in vivo [67]. Some of the dendritic action potentials were probably initiated in dendrites. In contrast, in the apical dendrites of layer 2/3 pyramidal neurons, no spontaneous or sensory-evoked dendritic 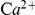 action potentials were observed [68], although moderate current injection into dendrites in vivo was able to induce dendritic 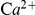 action potentials [69]. We therefore expect that moderate current injection induced in natural living conditions of an animal can evoke dendritic 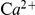 action potentials. Recently, the 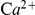 signal of a population of dendrites was imaged in vivo with a fiber optic mounted on a prism embedded in the cortex. The signal is expected to originate in the dendritic 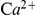 action potentials in apical tufts, which seems to correspond to the intensity of sensory stimulation [16]. We think these lines of evidence together strongly support the existence of the dendritic plateau potential existing in vivo.

### Possible functional implication: extension of Integral Time Windows

**Figure 9:**
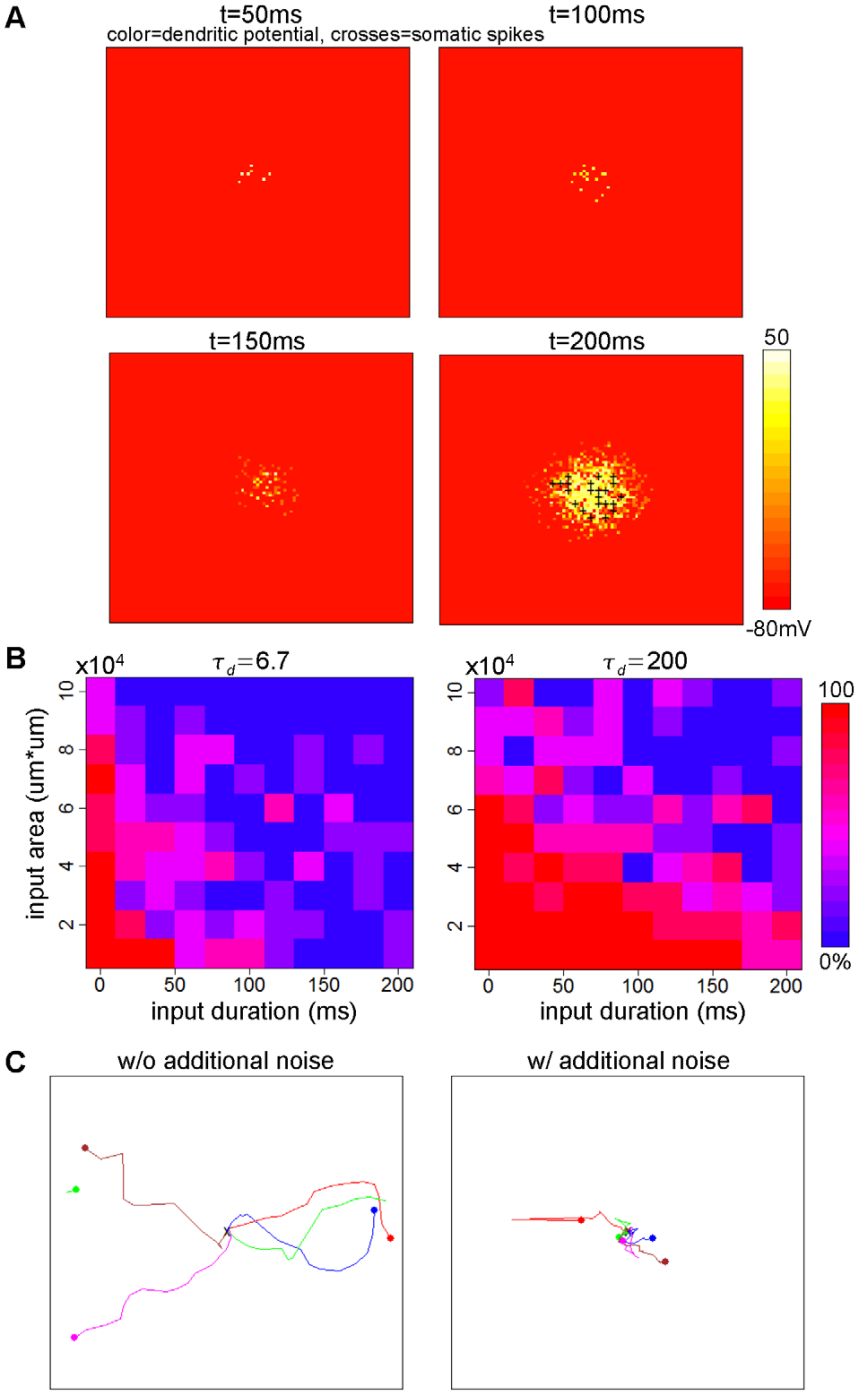
Expansion of the time window for spatio-temporal integration at network level. (A) The process of input integration. The history of the input to dendrites is stored as the dendritic plateaus and sufficient occurrences of dendritic plateaus cause the generation of clustered somatic action potentials (bump). The input duration and input area are 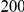 ms and 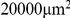, respectively. (B) The success rate of the integration to generate a bump is plotted versus the input duration and input area for the non-plateau (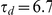 ms, left) and long-lasting plateau (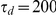 ms, right) cases. The long-lasting dendritic plateau expands the time window for the integration. (C) The trajectory of the center of the bumps with long-lasting dendritic plateau (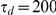 ms). External noisy currents after the generation of bumps can immobilize the bumps (right), while the bumps move in noiseless background conditions (left). The different colors indicate different trials.

We have hitherto examined the properties of the two-field model with the globally injected external inputs. However, since spatio-temporally structured external input is important in physiological information processing, we investigated the effects of the long-lasting dendritic plateau on the integration of spatio-temporally limited external inputs (Figure 9).

In these simulation runs, the external pulse-like inputs were injected into dendritic units, unlike previous simulations, where the inputs were continually injected into somatic units. The input intensity was adjusted so that a single input elicited a single dendritic plateau. The inputs were targeted at the disk-shaped area located at the center of the dendritic field. The times for receipt of the inputs were randomly distributed in a set period. We call the radius of the disk-shaped area the input radius, and call the duration of the period for the inputs the input duration. The values of the input radius and duration were systematically varied, while the number of total external inputs, which was 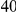, was unchanged throughout the simulation runs. Thus the large values of the input radius and duration came to sparse inputs. We then asked in what condition a persistent bump appeared, which we regarded as a sign of the spatio-temporal integration of the external inputs. Figure 9A shows how the integration developed. Initially, each dendritic unit received a single input pulse that elicited a single dendritic plateau. Although such an occurrence of a dendritic plateau alone did not elicit any somatic action potential, the currents lasted long when the value of 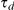 was large. The long-lasting currents gradually piled up at the soma and finally caused the somatic action potential to form a bump.

We investigated how the formation of a bump depends on the large values of the input radius and duration (Figure 9B). Naturally, the larger the values of the input radius and input duration are, the smaller the probability of the generation of a bump is. With the large 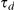, however, the integration could oppose the temporal sparseness of external inputs. Note that we set the value of 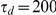 ms in the interest of computational time for the simulation, but experimental observations show that the value can be larger [10]. Hence, this suggests that the model involving a long-lasting dendritic plateau can integrate the external inputs on the behavioral timescale. In contrast, with small values of 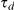, the time window for the integration is narrower, and the network detects only the coherent inputs. Thus, our network model can set a spatio-temporal time window to detect specific input signals.

As we observed previously, the noise could also immobilize a bump generated in this simulation run (Figure 9C).

### Implications for Short-Term Memory in the Visual Cortex

Ambiguous stimulus is a sensory stimulus eliciting multiple distinct perceptual interpretations. Representative examples are the Necker cube and Rubin's vase and faces. Psychophysical experiments have showed that one perceptual interpretation of such an object tends to be preserved after a short blank period intervening in the stimulus periods. This observation suggests the existence of short-term memory of the interpretation [70]. Recently, O'Herron and von der Heydt approached the problem of short-term memory of this kind by using visual stimuli involving figure-ground ambiguity [71]. They recorded neuron activity from area V2 of monkeys watching ambiguous figures. The firing of a specific neuron seemed to be associated with one interpretation of a figure. The firing of the neuron returned to the resting level during a blank intervening period between stimulus periods. Nevertheless, the same neuron started firing again when the same figure reappeared, meaning that the previous interpretation was recovered. The conceptual similarity of this phenomenon to the recovered bump after the inhibitory period (Figure 7) suggests the possibility that the recovered interpretation may be explained by the long-lasting dendritic dynamics.

## Methods

### Two-field model

The two-field model proposed in the present study consisted of somatic and dendritic fields, the dimension of which was 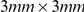. The somatic (dendritic) field was represented by squarely arrayed somatic (dendritic) units with an inter-unit distance of 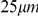 (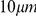).

A given dendritic unit is thought to receive massive innervation from nearby neurons. The innervation becomes more sparse with increased distance between the dendritic unit and the soma of a neuron (somatic unit) sending a synaptic terminal. Assume that a somatic unit is located at 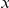 (2-dim position) and a dendritic unit is located at 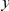 (2-dim position). We approximated the connection probability as the Gaussian function of somatodendritic distance, 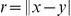: 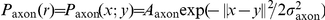, with 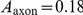 and 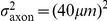 unless otherwise stated (Figure 1A). A given somatic unit is considered to have its own dendritic branches densely near the soma and sparsely far from the soma. Assume that another somatic unit is located at 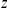 (2-dim position). We approximated the probability of finding a dendritic branch belonging to the soma as the Gaussian function of dendrosomatic distance, 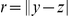: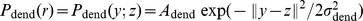, with 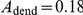 and 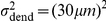 unless otherwise stated (Figure 1A). We determined all soma-to-dendrite and dendrite-to-soma couplings according to this probability, and we fixed these couplings before starting the simulation runs. Such an assumption of the Gaussian connection probabilities is supported by the experimental observation of the connectivity between cortical layer 2/3 pyramidal neurons [47], [48]. Our network model is the so-called field model [29], [30]. Therefore, as we explained in the Results, each dendritic unit is actually shared by neighboring somata. Such an assumption is considered reasonable when the synaptic input has spatial continuity, meaning that two dendritic branches next to each other should obtain similar input and so their local EPSP should be close.

As we explain in detail below, we adopted the Izhikevich model to describe the somatic membrane dynamics because it is a commonly used spiking neuron model due to its flexibility to reproduce spiking patterns of real neurons with a small computational cost. In describing the very slow dendritic plateau dynamics, we noted that the Morris-Lecar model worked better than the Izhikevich model for our purposes. Meanwhile, we used a rate based non-spiking model to describe the inhibitory neurons because the inhibitory unit represents a population of neurons and is not supposed to exhibit spiking behavior.

The dynamics of the somatic membrane potential of the 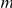
th somatic unit, 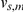, is described with a minimal extension (single auxiliary variable) of the integrate-and-fire model (Izhikevich model) [51]:
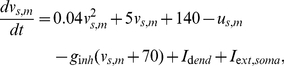
(1)

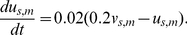
(2)

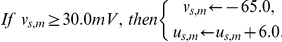
Auxiliary variable, 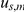, gives an after-hyperpolarizing current to the right hand side of the Eq. (1). Of the two input current sources to the soma, 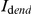 represents the current from all of its dendritic branches, which we consider originates in the intralaminar (within layer 2/3) recurrent synaptic connections, while the other source, 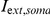, is supposed to represent the extralaminar (layer 4 or thalamus) input combined with the noisy background input.

The dynamics of the membrane potential of 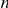
th dendritic unit, 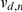, is described by the Morris-Lecar formalism [52] with its after-hyperpolarization truncated (explained later):
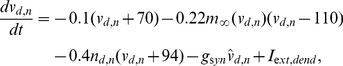
(3)

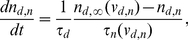
(4)

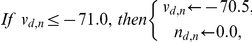
with
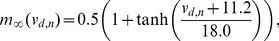
(5)

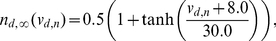
(6)

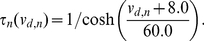
(7)The rescaled dendritic potential, 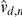, appearing in Eq. (3) is defined later. A dendritic unit represents a tiny part of a dendritic tree. Its local membrane potential is depolarized whenever an innervating neuron fires a somatic spike. The trajectory of 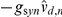 is determined by the first order dynamics, 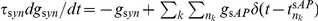 with 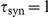 ms.

Another constant, 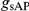, is set to 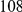 (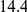) for the simulation runs shown in Figures 2, 3, 4 and 9 (Figures 5, 6, 7, 8 and S3). Here, 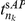 denotes 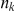 th spike time of soma 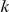 that synapses on the dendritic unit described above. Current involving 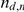 contributes an intrinsic hyperpolarizing current that balances the excitatory drive. Therefore, in this paper we refer to this as the dendritic 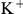 conductance. Similarly, 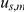 plays an inhibitory role, and then we call it somatic 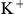.

A current from a dendritic branch to the soma should be proportional to the voltage difference between the dendritic branch and the soma. Because the current is the summation of currents over all the dendritic branches, we have 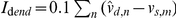. To moderate the effect of the dendritic plateau, the dendritic potentials for synaptic and dendritic currents were set to 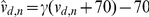. The constant, 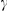, was set to 0.73 except in Figure 9 where 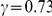 and 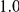 for 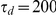 and 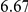, respectively.

Our model has a one-way signal flow from the dendritic units to the somatic unit. A passive current flow from the soma to dendrites is safely neglected because of the large input impedance of the dendritic compartments compared to that of the soma. However, the active dendritic flow carried by the backpropagating action potential has been widely observed in vitro. An important question is therefore whether such active dendritic currents could invalidate our scenario in vivo. The answer to the question is presently unavailable because of the lack of sufficient in vivo observations. According to the in vitro observations, the backpropagating action potential was reported to enhance the generation of dendritic action potential [5], [72]. The presence of the influence of the backpropagating action potential itself looks inconsistent with our simplifying assumption. However, the enhancement of the dendritic potential generation goes well with our scenario. In another case, a shunting inhibition caused by backpropagating action potential was reported to be limited for the active dendritic potential via NMDA-current [73].

Considering the current understanding, we believe the theoretical argument based on the simplifying assumption to be a useful first step toward a full understanding of the role of the active dendritic potential in a network setting.

The Morris-Lecar model used in the present study is meant to be a tool describing the dendritic plateau observed. The model in fact works very well in producing the plateau-like lasting elevation of the voltage with a clear all-or-none nature for high values of 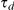 (Figure 1B). The model, however, also produces too deep a hyperpolarization after the plateau, which is different from what is observed in the dendritic dynamics [53]. To prevent such unnecessary hyperpolarization from appearing, we set the condition indicated by the “if” clause.

We consider here that a collection of inhibitory neurons, that we model with a single unit, play a role in adjusting the total activation level of the network in a reasonable range. Inhibitory synaptic conductance, 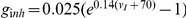 is assumed to suppress the somatic activity of excitatory neurons through the fifth term on the right-hand side of Eq. (1). The membrane potential, 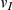, of the inhibitory unit is governed by conventional first order dynamics, 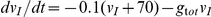, the driving term of which sums the network activity of pyramidal neurons with equation, 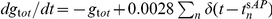. Here 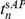 represents the somatic spike time of all the neurons in the network.

All the membrane potentials in these equations are in units of millivolts, all currents are in units of picoamperes, and all conductances are in units of mS/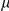
F. The units of the coefficients in the equations are given to ensure the right dimensionality between the left and right sides. We numerically solved the model with the 4th order Runge-Kutta method at a temporal resolution of 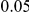 ms by Intel C++ Compiler.

### External current injection

In our model, three types of external input current were used: a constant one, a noisy one and an inhibitory one. These input currents were injected into the somatic unit (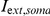) in all the simulations except those leading to Figure 9 where the short pulses of constant currents were injected into the dendritic units (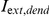). The constant input current was defined by its amplitude, 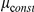. The noisy input current was the Gaussian white noise defined by its variance 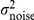. Such noisy current was generated numerically in the standard manner [74]. As an inhibitory input current, we used 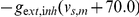 with 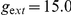, which was strong enough completely to prevent the somatic action potential from occurring.

In the simulation runs leading to Figure 2, a constant input current was continually injected throughout the simulation run, while in the runs leading to Figures 3, 4 and 5, noisy input current was continually injected throughout the run. In the runs leading to Figure 6A, constant input current was given for 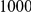 ms, then the current switched to a noisy one, which lasted for 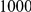 ms. Finally a transient inhibitory input current was injected between 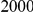 ms and 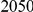 ms. In the simulation run leading to Figure 6B, noisy currents were injected throughout the simulation run. At 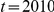 ms, transient (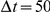 ms) excitatory input was applied along a thin linear area over the stationary bump followed immediately by another transient (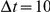
ms) excitatory input along a thin linear area adjacent to the previously simulated area. In the runs leading to Figure 7, constant input current was injected for the first 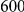 ms, then the input current was switched to an inhibitory one, which continued for several tens of milliseconds (different durations in 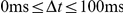 were tested). Finally, noisy input current was injected until the end of a simulation run.

In the runs leading to Figure 9B brief (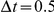 ms) pulses of constant input current were injected into the dendritic units with their amplitudes of 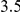 or 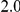 and their 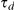 of the 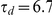 ms or 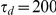 ms, respectively in the two cases displayed. Each input was strong enough to induce a single dendritic plateau. For each input, the input time and inputted dendritic unit were uniformly randomly selected from the temporal period, the length of which was described by the input duration, and the spatial region, the shape of which was disk-like. The radius was described by the input radius. The number of total inputs was 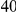.

### Measure of immobility

The inner product measures how a snapshot (defined exactly below) of the somatic spiking activity at 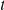 and that at 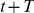 are similar. To calculate the snapshot, we first count the number of somatic spikes occurring within a 10 ms time window at some time point for each soma, which gives us a representation of the somatic spiking activity in a 120×120 matrix. To see the difference in this matrix at different time points, the simplest way is to take an inner product of the matrices corresponding to the different time points. However, the disadvantage of the simple inner product is that all the 120×120 components are treated independently in its calculation and geometrical information, such as that the soma at (101,50) is next to the one at (100,51), is not taken into account. To take the information into account, we applied the spatial Gaussian filter, 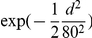, first. We call the resultant matrix a snapshot of the somatic spiking activity. We then calculate the inner product of the snapshots at 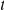 and 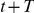 and average the products over 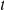 (sliding window) and over trials to smooth the temporal fluctuations. The resultant number quantifies how the somatic spiking activity is similar between time points separated by 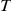. The sliding-window averaging makes sense only when the network activity is stationary. No averaging was taken for Figure 7, where no stationarity was expected. The baseline of an inner product is determined as the averaged inner products of the snapshots taken from different trials in the same condition. We subtract the baseline from the inner product. Then the inner product peaks at the zero time lag, and we normalize the peak value to be unity. Each inner product shown in the Figures and used to calculate 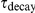 (described below) is already averaged and normalized.

Generally, the inner product decreases with an increase in the time lag, 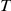. A decaying time constant of the inner product, 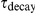, serves as a good measure of the immobility of the activity (not necessarily the bump activity). The inner product is fitted with an exponential function to obtain the value of 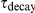. Negative values which come from tiny errors of the normalization but do not seriously affect our results were neglected in the exponential fitting.

### Clustering index of somatic action potential

The clustering index of somatic action potential measures the degree of the spatial clustering. To calculate the clustering index, we divided the somatic field into a square cell of four-by-four somatic units. We then counted the number of spiking somata in each cell in a given time bin, the width of which was 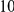 ms. Then we calculated the mean and standard deviation over all the compartments in a given time bin to obtain the coefficient of variance (CV) by dividing the standard deviation by the mean. Finally, we averaged the CVs over the simulation time, which gives the clustering index. Clustering indices of somatic and dendritic 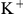 conductances (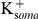 and 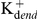, respectively) *summed* the values of 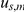 or 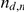 for each cell consisting of eight-by-eight somatic units or ten-by-ten dendritic units. To calculate the clustering indices of somatic and dendritic 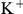 conductances (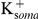 and 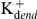, respectively), we *summed* the values of 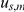 or 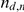, unlike in the case of the clustering index of somatic action potentials, where we counted the spiking somata for each cell consisting of eight-by-eight somatic units or ten-by-ten dendritic units. The remaining calculations are the same for somatic action potentials. Before summation, we normalized all of the data to make them range between zero and one. For the sake of convenience, we cite a textbook for the spatial analysis [75].

### Distribution of 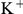 current

In order to measure the potassium current distribution, we delineated a bump in the following manner. We first smoothed the spatial distribution of the values of 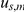 and 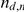 to obtain a contour with the Kriging method [75], and rescaled these variables so that each of them ranged between zero and one. We then removed the areas on the somatic (dendritic) field for which 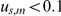 (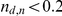). This procedure discards areas containing noisy potassium current fluctuations and leaves only a significantly thick bunch of potassium currents, which we call the core. Next, we calculated the center of gravity of the core and calculated the two principal components of the potassium current variance in the core. We regarded the center of gravity as the origin of the axes of the principle components. Finally, we calculated the skewness of the distributions of the values of 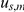 or 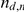 along the axes of the second principle component.

## Supporting Information

Figure S1
**Tolerance of a bump against noise becomes higher as the dendritic dynamics slows.** (A) The decay of the inner product with time lag becomes slow with the increase of the dendritic time constant, 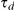. It means that the slow dendrite increases the capability of protection of the bump from breaking up by noisy inputs. The presented curves are obtained by temporal and trial average of the time lag-dependent inner products expressed by 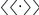. (B) The panel shows the dependence of the decay time constant calculated by curves of the inner product on 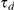. 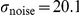 pA.(TIF)

Figure S2
**Re-quantification of the invariability of the bumps.** The invariability of the bumps were quantified by the times for the 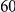
% reduction of the initial values. *A*, *B*, *C* and *D* correspond to the Figures 2D, 3B, 4B and S3A, respectively.(TIF)

Figure S3
**Simulation runs with a small value of EPSP.** Simulations with the EPSP size 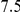 time smaller than that used in Figure 3. The time constant of dendritic dynamics is set to 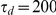 ms. (A) The decay time constant obtained by the exponential fitting of the inner products is shown in *B*. The results look similar to those obtained with the larger value of EPSP (Figure 3). (inset) The clear bump in the network activity is observed at 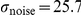 pA. (B) Inner products of somatic activity patterns for different values of noise intensity, 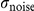. (C) The clustering of somatic action potentials declines with the increasing noise intensity, 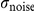. (D) The destruction-corrected degree of immobilization as a product of *A* and *C* that are scaled to make those range be unity shows the clear high-value zone, which means that strong noise immobilizes the bump activity without breaking up the bump.(TIF)

Table S1 **Summary of the relationship between the observed bump modes and the noise and dendritic conditions.**
(TIF)

Video S1
**Switching of a bump mode from mobile to immobile in the noisy condition.** A moving bump born in the noiseless background (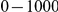 ms) keeps moving in the noisy background (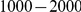 ms). However, strong global inhibition (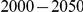 ms) switches a bump to immobile mode. The bump movement is restricted to the linear strip for the best illustration of the phenomenon. For more detail, see main text and the caption of Figure 6A.(MOV)

Video S2
**Switching of a bump mode from immobile to mobile in the noisy condition.** In the noisy background condition, transient strong excitatory input along a horizontal line the center of the bump (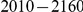 ms) followed by the continuing short-term strong excitatory input along a horizontal line adjacent to the previously simulated line (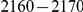 ms) induces the bump to switch from immobile to mobile mode. The bump movement is restricted to the linear strip for the best illustration of the phenomenon. For more detail, see main text and the caption of Figure 6B.(MOV)

Video S3
**Searching and fixation by the present mechanism.** First, a bump moves under the noisy condition (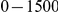 ms). When the bump reaches the location where the something worth is (black square), the transient global inhibition comes (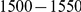 ms) and switches the bump to immobile mode. For more detail, see main text and the caption of Figure 8.(MOV)
