# Supplementary figures and images for: Dendritic Slow Dynamics Enables Localized Cortical Activity to Switch between Mobile and Immobile Modes with Noisy Background Input
Source: PLoS One. 2011 Sep 8;6(9):e24007. doi: 10.1371/journal.pone.0024007 (PMC3169558; doi:10.1371/journal.pone.0024007)

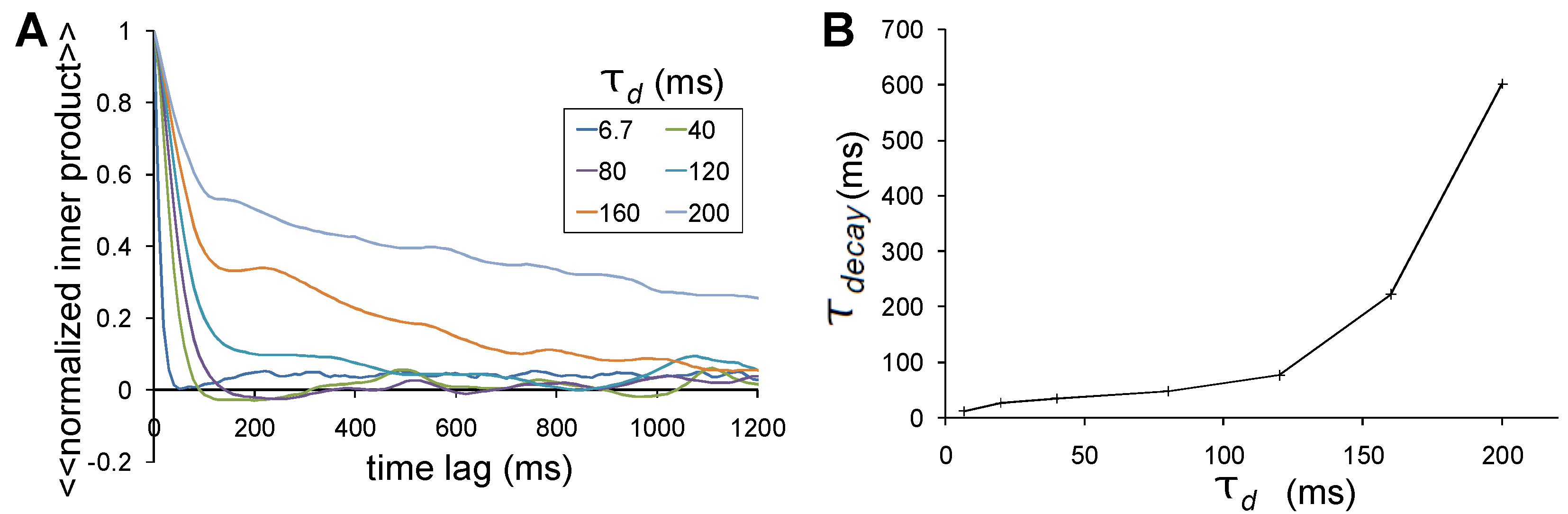

Supplement: Figure S1 — Tolerance of a bump against noise becomes higher as the dendritic dynamics slows. (A) The decay of the inner product with time lag becomes slow with the increase of the dendritic time constant, . It means that the slow dendrite increases the capability of protection of the bump from breaking up by noisy inputs. The presented curves are obtained by temporal and trial average of the time lag-dependent inner products expressed by . (B) The panel shows the dependence of the decay time constant calculated by curves of the inner product on . pA. (TIF) [file pone.0024007.s001.tif]

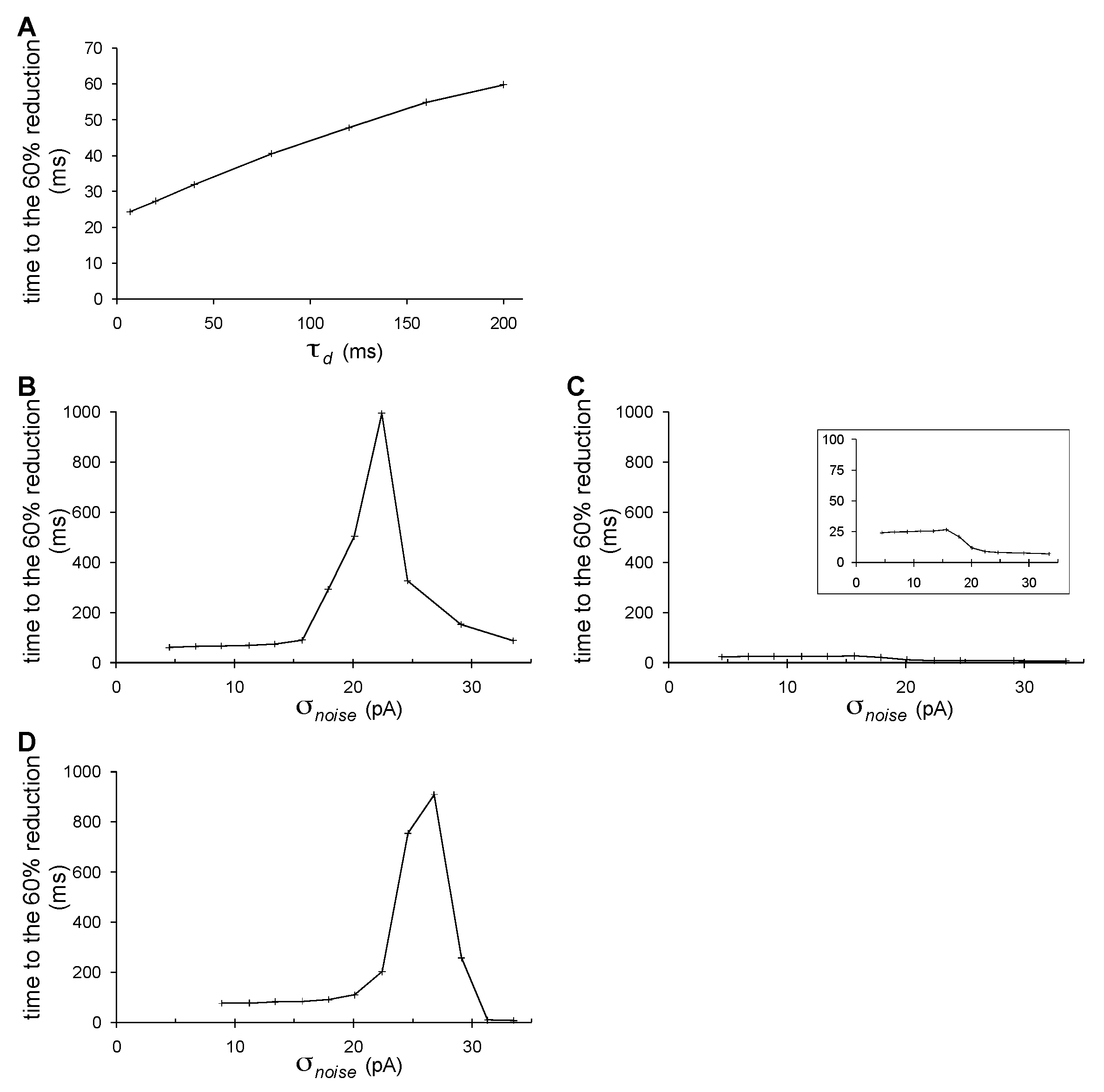

Supplement: Figure S2 — Re-quantification of the invariability of the bumps. The invariability of the bumps were quantified by the times for the % reduction of the initial values. A, B, C and D correspond to the Figures 2D, 3B, 4B and S3A, respectively. (TIF) [file pone.0024007.s002.tif]

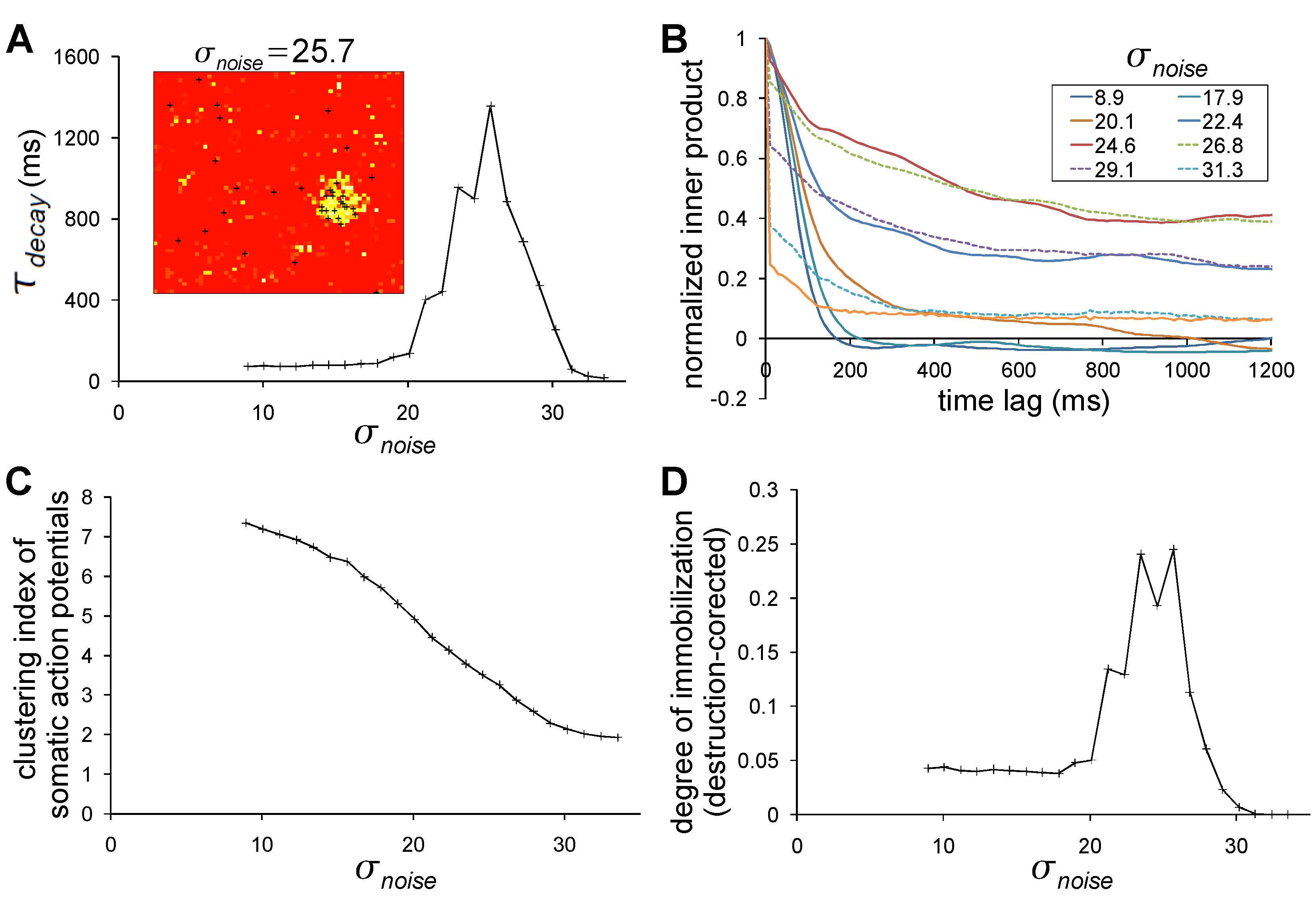

Supplement: Figure S3 — Simulation runs with a small value of EPSP. Simulations with the EPSP size time smaller than that used in Figure 3. The time constant of dendritic dynamics is set to ms. (A) The decay time constant obtained by the exponential fitting of the inner products is shown in B. The results look similar to those obtained with the larger value of EPSP (Figure 3). (inset) The clear bump in the network activity is observed at pA. (B) Inner products of somatic activity patterns for different values of noise intensity, . (C) The clustering of somatic action potentials declines with the increasing noise intensity, . (D) The destruction-corrected degree of immobilization as a product of A and C that are scaled to make those range be unity shows the clear high-value zone, which means that strong noise immobilizes the bump activity without breaking up the bump. (TIF) [file pone.0024007.s003.tif]

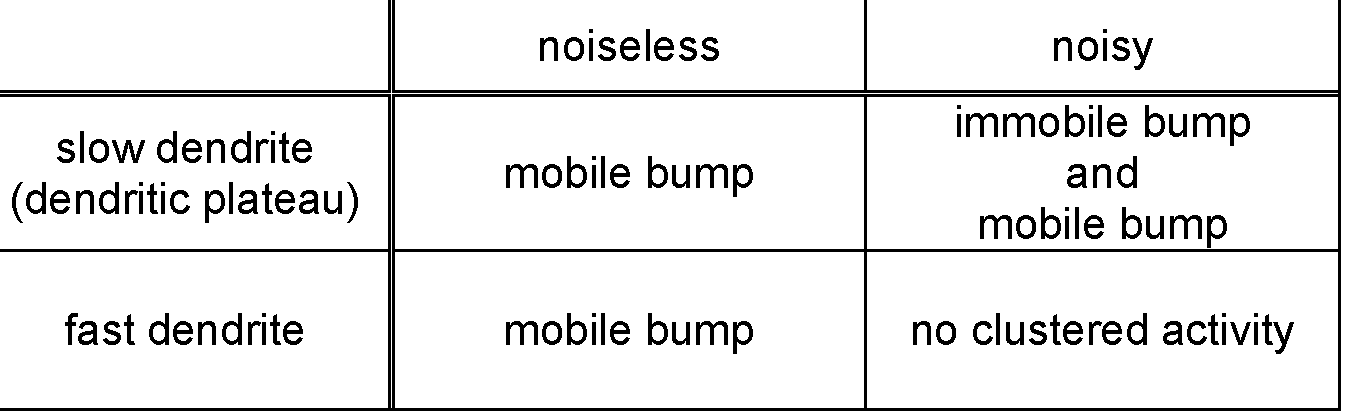

Supplement: Table S1 — Summary of the relationship between the observed bump modes and the noise and dendritic conditions. (TIF) [file pone.0024007.s004.tif]
